# Cord Blood Thyroid Hormones and Neurodevelopment in 2-Year-Old Boys and Girls

**DOI:** 10.3389/fnut.2021.773965

**Published:** 2021-12-20

**Authors:** Pianpian Fan, Yuanzhi Chen, Zhong-Cheng Luo, Lixiao Shen, Weiye Wang, Zhiwei Liu, Jun Zhang, Fengxiu Ouyang

**Affiliations:** ^1^Ministry of Education and Shanghai Key Laboratory of Children's Environmental Health, Xinhua Hospital, Shanghai Jiao Tong University School of Medicine, Shanghai, China; ^2^Department of Obstetrics and Gynecology, Prosserman Centre for Population Health Research, Lunenfeld-Tanenbaum Research Institute, Mount Sinai Hospital, Institute of Health Policy, Management and Evaluation, University of Toronto, Toronto, ON, Canada; ^3^Department of Neonatology, International Peace Maternity and Child Health Hospital, Shanghai Jiao Tong University School of Medicine, Shanghai, China

**Keywords:** thyroid hormones, neurodevelopment, cord blood, ASQ-3, infancy, boys and girls

## Abstract

**Objective:** Thyroid hormones are essential for neurodevelopment in early life. However, the impact of mild alterations in neonatal thyroid hormones on infant neurodevelopment and its sex dimorphism is unclear. We aimed to assess whether mild variations in neonatal thyroid hormones of term-born newborns with maternal euthyroid are related to neurodevelopment in 2-year-old boys and girls.

**Methods:** This study used data from 452 singleton term-born infants of mothers with normal thyroid function in Shanghai, China, and their follow-up measure at the age of 2 years. Cord serum concentrations of free thyroxine (FT4), free triiodothyronine (FT3), thyroid-stimulating hormone (TSH), and thyroid peroxidase antibody (TPOAb) were measured by chemiluminescent microparticle immunoassays and classified into three groups: the low (1st, Q1), middle (2nd−4th, Q2–Q4), and high (5th, Q5) quintiles. Neurodevelopment indices were assessed using the Ages and Stages Questionnaire, third edition (ASQ-3), at 24 months of age.

**Results:** Compared to infants with thyroid hormones in the middle (Q2–Q4), boys with FT4 in the lowest quintile had 5.08 (95% CI: 1.37, 8.78) points lower scores in the communication domain, 3.25 (0.25,6.25) points lower scores in the fine motor domain, and 3.84 (0.04, 7.64) points lower scores in the personal-social domain, respectively. Boys with FT3 in the highest quintile had 4.46 (0.81, 8.11) points increase in the personal-social domain. These associations were not observed in girls. No associations were observed between cord blood serum TSH and ASQ-assessed neurodevelopment in the boys or the girls.

**Conclusions:** Mild alterations in thyroid hormones of newborns were associated adversely with neurodevelopment in boys, suggesting the importance of optimal thyroid hormone status for neurodevelopment in early life.

## Introduction

An adequate supply of thyroid hormones is essential for healthy neurodevelopment *in utero* and during infancy (the first 2 years of postnatal life) (1, 2). During early gestation, fetal thyroid hormones are of maternal origin, and from mid gestation onward, fetal thyroid gland begins to secrete thyroxine (T4) and triiodothyronine (T3) under the control of the hypothalamic-pituitary-thyroid axis, and the thyroid hormone axis becomes fully functional around the time of term birth (3). In early postnatal period, continuing maturation of the brain is dependent on neonatal thyroid hormones (2). The developing brain may be susceptible to even mild thyroid hormone deficiency (4). However, it is unclear whether mild variations in neonatal thyroid hormones may affect subsequent neurodevelopment during infancy.

Neonatal levels of thyroid hormones have been related to gestational brain development as well as postnatal neurodevelopment (5, 6). Studies in recent decades have showed that variations in the lower and higher ranges of neonatal thyroid hormone levels may be associated with neurodevelopment, but data are limited and contradictory (7). Some studies showed that higher neonatal thyroid-stimulating hormone (TSH) levels, although below normal screening thresholds, were associated with poorer cognitive or behavioral development (8–11), while no association was reported in other studies (12–14) or the association might be dependent on TSH levels (15). Lower free thyroxine (FT4) concentrations have been related to worse neurodevelopment in preterm babies (6, 16, 17). However, there were also reports of no significant associations (18–21) or age-dependent associations (22). There are several randomized controlled trials (RCTs) addressing the effect of thyroid hormone supplementation during infancy on neurodevelopment and reporting that T4 supplementation does not improve mental or motor development in preterm infants (23) and in infants with Down syndrome (24). It remains unclear whether mild variations in neonatal thyroid hormones, within normal ranges, may affect neurodevelopment during infancy. In addition, the brain undergoes substantial organization and sexual differentiation during infancy (25), and it is unclear whether sex has an impact on the association between neonatal thyroid hormones and neurodevelopment.

Cord blood thyroid hormones levels are proxy for *in utero* thyroid functional status (26). In this prospective cohort study, we tested the hypothesis that relatively low cord blood FT4 and free triiodothyronine (FT3) levels in term-born infants of euthyroid mothers may reflect suboptimum thyroid function during gestation and may have an impact on neurodevelopment during infancy.

## Methods

### Study Design and Participants

The study participants were from the Shanghai Obesity and Allergy Cohort described previously (27). Briefly, between 2012 and 2013, women with singleton pregnancy at late trimester (close to delivery) were recruited in two tertiary hospitals in Shanghai, China. The study was approved by the Medical Ethics Committee of Xinhua Hospital, Shanghai Jiao Tong University School of Medicine. Written informed consent was obtained from the parents of the infants.

In this cohort, there were 1,051 mother-infant pairs with data on cord blood serum FT4, FT3, TSH, or thyroid peroxidase antibody (TPOAb). Neurodevelopment assessments were completed in 494 infants at 24 months of age. We excluded infants of mothers with thyroid diseases (hyperthyroidism, *n* = 5; hypothyroidism, *n* = 12) and syphilis (*n* = 3), and infants with artificial fertilization (*n* = 10), and preterm births (*n* = 14), among whom two had both artificial fertilization and preterm births. Thus, the final study sample included 452 mother-infant pairs. No infants included were diagnosed with congenital hypothyroidism or congenital anomalies.

### Cord Serum FT4, FT3, TSH, and TPOAb Assays

Immediately after delivery, cord blood sample was collected. Aliquots of serum samples were stored at −80°C until the assays. Cord blood serum FT4, FT3, TSH, and TPOAb concentrations were measured by chemiluminescent microparticle immunoassays using the ARCHITECT System (Abbott Laboratories, Abbott Park, IL, United States) in the clinical biochemistry laboratory of Shanghai International Peace Maternity and Child Health Hospital of Chinese Welfare Foundation; the laboratory is certified by the National Accreditation Board of China. QA/QC procedures were performed for all the assays in accordance with the system's instructions. Inter- and intra assay coefficients of variation were 2.5–6.3 and 3.5% for FT4, 5.9 and 5–5.1% for FT3, and 2.5–4.1 and 2.2–2.9% for TSH. The limits of detection (LOD) for FT4, FT3, TSH, and TPOAb were 5.15 pmol/L, 1.54 pmol/L, 0.01 mIU/L, and 0.5 IU/ml, respectively. The cross-reactivity was 0.002% between the T3 and T4 assays for FT4 > 1,000,000 pg/ml, and 0.0035% for FT3 > 12,000 ng/dl. Therefore, the assays showed virtually no cross-reactivity between FT3 and FT4. The TPOAb positive was defined as TPOAb ≥5.61 IU/ml (28).

### Neurodevelopment Assessment

Neurodevelopment during infancy was assessed (at 24 months of age) using the Ages and Stages Questionnaires, third edition (ASQ-3) (29). This standardized developmental screening tool is designed for children 1–66 months of age. Each of the age-specific questionnaires contains 30 items over five domains: communication, gross motor, fine motor, problem solving, and personal-social skills. The answer to each item is one of the following: “yes,” “sometimes,” and “not yet” and was scored 10, 5, and 0 points, respectively. Scores in each subscale ranged from 0 to 60. Suspected developmental delay was indicated by a domain score of 1–2 SD below the mean, while developmental delay was defined as domain score > 2 SD below the mean using the reference norms (29). More than 92% of the questionnaires were completed by the parents of the infants. The high inter-rater reliability of the ASQ-3 questionnaires was confirmed in a Chinese population, with a coefficient of between 0.8 and 0.84 (30, 31).

### Maternal and Infant Factors

Maternal and infant characteristics included maternal age, education, infant sex, gestational age at birth, birth weight, 5-min Apgar score, age at ASQ-3 assessment, infant feeding pattern during the first 6 months of life, and infant exposure to passive smoking in the first 2 years of postnatal life. There were three infant feeding categories: (1) exclusive breast-feeding, (2) formula feeding, and (3) mixed feeding (32). Birth weight for gestational age was defined according to the Chinese birth weight references for male and female newborns separately (33), and categorized as small for gestational age (SGA, <10th percentile), appropriate for gestational age (AGA, 10th−90th percentile), and large for gestational age (LGA, >90th percentile).

### Statistical Analysis

Student's *t*-test and Chi-square test were performed to assess the differences in continuous and categorical variables. Considering that cord serum levels of thyroid hormones differ between infants born by vaginal and cesarean section deliveries (34), and the non-linear associations with neurodevelopment, FT4, FT3, and TSH levels were categorized into mode-of-delivery specific quintiles in comparisons of neuro-developmental outcomes. For cord serum thyroid hormone, the middle 60% (2nd−4th quintiles, Q2–Q4) was set as the reference group to analyze the effects of low (1st quintile, Q1) or high (5th quintile, Q5) levels on neurodevelopment. Associations between neonatal thyroid hormone levels and ASQ-3 scores in boys and girls were assessed in generalized linear models. Adjusted analyses were controlled for maternal age (<30, 30–34, ≥35 years), education (high school or lower, college/university), birth weight for gestational age (SGA, AGA, LGA), infant feeding pattern during the first 6 months of life (exclusive breast-feeding, formula feeding, mixed feeding), any passive smoking in the first 2 years of life (yes, no), and age at ASQ-3 assessment. In multivariable regression models, we coded the missing values as a separate category for the covariate:infant feeding pattern during the first 6 months. *P* <0.05 were considered statistically significant. Data analyses were conducted using the Statistical Analysis System (SAS), version 9.4 (SAS Institute, Inc, Cary, NC, United States).

## Results

### Study Population Characteristics

Average mother age was 29 ± 3.4 (mean ± SD) years. About 97.8% of the mothers were Han Chinese, 86.3% had college or university education, and 76.1% of the infants were cesarean section deliveries. All the newborns were full term and had normal 5-min Apgar score (≥8). Average age at ASQ-3 assessment was 24.17 ± 0.71 months. The prevalence of exclusive breast-feeding in the first 6 months of life was 38.6%. Exposure to passive smoking in the first 2 years of postnatal life was 58.6% in the infants. The boys had higher birth weight and cord blood TSH concentration than the girls (Table 1).

**Table 1:** Maternal and infant characteristics of 452 singleton term-born infants of mothers with normal thyroid function during pregnancy in a birth cohort from Shanghai, China.

**Characteristic**	**Boys**	**Girls**	***p*-value**
	**(*n* = 237)**	**(*n* = 215)**	
Maternal age (years)			0.142
<30	152 (64.1)	132 (61.7)	
30–34	74 (31.2)	62 (29.0)	
≥35	11 (4.7)	20 (9.3)	
Maternal education			0.217
High school or lower	28 (11.8)	34 (15.8)	
College/university	209 (88.2)	181 (84.2)	
Maternal passive smoking during pregnancy			0.170
No	171 (72.5)	143 (66.5)	
Yes	65 (27.5)	72 (33.5)	
Mode of delivery			0.762
Vaginal delivery	58 (24.5)	50 (23.3)	
Cesarean section	179 (75.5)	165 (76.7)	
Birth weight for gestational age			0.279
SGA	7 (3.0)	13 (6.0)	
AGA	201 (84.8)	176 (81.9)	
LGA	29 (12.2)	26 (12.1)	
Infant feeding pattern in the first 6 months			0.060
Formula feeding	15 (7.0)	27 (14.0)	
Exclusive breast-feeding	83 (38.8)	74 (38.3)	
Mixed feeding	116 (54.2)	92 (47.7)	
Infant exposure to passive smoking during the first 2 years			0.236
No	92 (38.8)	94 (44.3)	
Yes	145 (61.2)	118 (55.7)	
Cord blood serum TPOAb			0.223
Negative	205 (87.6)	196 (91.2)	
Positive	29 (12.4)	19 (8.8)	
Gestational age at birth (weeks)	39.05 ± 0.97	39.05 ± 0.93	0.963
Birth weight (g)	3,484.6 ± 406.3	3,358.0 ± 421.0	0.001
Cord blood serum thyroid hormones			
FT4 (pmol/L)	13.26 ± 1.66	13.39 ± 1.43	0.362
FT3 (pmol/L)	1.98 ± 1.20	1.92 ± 0.77	0.521
TSH (mIU/L)	6.60 ± 4.89	5.72 ± 3.22	0.024
Infant age at ASQ-3 assessment (months)	24.15 ± 0.67	24.19 ± 0.76	0.522

*SGA, small for gestational age; AGA, appropriate for gestational age; LGA, large for gestational age; TPOAb, thyroid peroxidase antibody. TPOAb positive: ≥ 5.61 IU/ml; FT4, free thyroxine; FT3, free triiodothyronine; TSH, thyroid-stimulating hormone.*

*Data are shown as N (%) or mean ± standard deviation (SD)*.

### Infant Neurodevelopment at 24 Months of Age

The mean scores were 43–54 for the communication, gross motor, fine motor, problem solving, and personal-social domains at 24 months of age for the boys, and 48–56 for the girls (Table 2). Compared with the girls, the boys had a higher proportion of delay and suspected delay in the communication (17.3 vs. 7.9% for boy vs. girl, *p* = 0.003), and personal-social (19 vs. 5.1%, *p* < 0.0001; Table 2) domains.

**Table 2:** Ages and stages questionnaire, third edition (ASQ-3) scores and prevalence of developmental delays in boys and girls at 24 months of age.

**Domain**	**Boys (*****n*** **=** **237)**				**Girls (*****n*** **=** **215)**				***p*-value^**a**^**	***p*-value^**b**^**
	**Mean± SD**	**Normal**	**Suspected delay**	**Delay**	**Mean ± SD**	**Normal**	**Suspected delay**	**Delay**		
		***n* (%)**	***n* (%)**	***n* (%)**		***n* (%)**	***n* (%)**	***n* (%)**		
Communication	51.86 ± 10.69	196 (82.7)	23 (9.7)	18 (7.6)	56.09 ± 7.72	198 (92.1)	12 (5.6)	5 (2.3)	<0.0001	0.003
Gross motor	54.37 ± 7.44	222 (93.7)	6 (2.5)	9 (3.8)	54.69 ± 7.92	199 (92.5)	7 (3.3)	9 (4.2)	0.652	0.640
Fine motor	50.69 ± 8.94	191 (80.6)	39 (16.5)	7 (2.9)	51.84 ± 7.79	185 (86.0)	27 (12.6)	3 (1.4)	0.145	0.121
Problem solving	49.89 ± 8.41	224 (94.5)	9 (3.8)	4 (1.7)	51.58 ± 8.33	201 (93.5)	12 (5.6)	2 (0.9)	0.033	0.646
Personal-social	43.35 ± 11.19	192 (81.0)	34 (14.8)	10 (4.2)	48.09 ± 9.89	204 (94.9)	7 (3.2)	4 (1.9)	<0.0001	<0.0001

*^a^Student's t test was performed to test the mean differences between boys and girls.*

*^b^Chi-square test was performed to assess the differences in categorical variables between boys and girls with suspected delay and delay combined*.

### Cord Blood Thyroid Hormones and Infant Neurodevelopment

Cord serum FT4, FT3, and TSH concentrations were categorized into mode-of-delivery-specific quintiles (Supplementary Table 1). Low cord serum FT4 levels were associated with lower ASQ-3 scores. Compared to the boys with FT4 in the middle 60%, those with FT4 in the lowest quintile (Q1) had 5.08 (95% CI: 1.37, 8.78) points lower scores in the communication domain, 3.25 (0.25, 6.25) points lower scores in the fine motor domain, and 3.84 (0.04, 7.64) points lower scores in the personal-social domain. Compared to the boys with cord serum FT3 in the middle 60%, those with FT3 in the highest quintile (Q5) had 4.46 (0.81, 8.11) points higher scores in the personal-social domain (Table 3).

**Table 3:** Associations between cord blood thyroid hormones and ASQ-3 scores in boys at 24 months of age.

**Thyroid related hormones**	**Communication**		**Gross motor**		**Fine motor**		**Problem solving**		**Personal-social**	
	**Mean±SD**	**Adjusted**	**Mean±SD**	**Adjusted**	**Mean±SD**	**Adjusted**	**Mean±SD**	**Adjusted**	**Mean±SD**	**Adjusted**
		**β (95% CI)**		**β (95% CI)**		**β (95% CI)**		**β (95% CI)**		**β (95% CI)**
**FT4 (pmol/L)**										
1st quintile (*n* = 44)	47.73 ± 11.88	−5.08 (−8.78, −1.37)**	51.82 ± 9.09	−2.32 (−4.78, 0.13)	47.34 ± 11.42	−3.25 (−6.25, −0.25)*	48.18 ± 10.24	−2.07 (−5.02, 0.89)	39.89 ± 13.01	−3.84 (−7.64, −0.04)*
Mid 3 quintiles (*n* = 150)	52.50 ± 10.72	Ref	54.47 ± 7.47	Ref	50.93 ± 8.28	Ref	50.27 ± 7.68	Ref	43.87 ± 10.58	Ref
5th quintile (*n* = 43)	53.84 ± 8.15	1.17 (−2.54, 4.88)	56.63 ± 4.04	2.27 (−0.19, 4.73)	53.26 ± 7.31	1.93 (−1.07, 4.94)	50.35 ± 8.76	0.08 (−2.88, 3.04)	45.12 ± 10.83	0.63 (−3.19, 4.44)
**FT3 (pmol/L)**										
1st quintile (*n* = 49)	53.98 ± 9.35	3.59 (−0.01, 7.19)	54.39 ± 7.82	0.30 (−2.14, 2.74)	51.33 ± 9.34	0.84 (−2.13, 3.81)	51.12 ± 8.80	2.28 (−0.60, 5.15)	45.18 ± 10.34	3.62 (−0.06, 7.29)
Mid 3 quintiles (*n* = 137)	50.58 ± 11.12	Ref	53.98 ± 7.79	Ref	50.12 ± 8.70	Ref	48.94 ± 8.77	Ref	41.50 ± 11.46	Ref
5th quintile (*n* = 48)	53.85 ± 9.96	3.32 (−0.26, 6.90)	55.21 ± 6.10	0.88 (−1.55, 3.31)	51.60 ± 9.39	1.21 (−1.74, 4.16)	51.15 ± 6.78	2.08 (−0.77, 4.94)	46.38 ± 10.48	4.46 (0.81, 8.11)*
**TSH (mIU/L)**										
1st quintile (*n* = 48)	52.60 ± 9.28	1.40 (−2.24, 5.04)	53.75 ± 7.89	−0.76 (−3.18, 1.66)	51.67 ± 7.67	1.73 (−1.21, 4.67)	50.83 ± 8.46	1.94 (−0.90, 4.78)	44.67 ± 11.62	2.21 (−1.50, 5.91)
Mid 3 quintiles (*n* = 132)	51.10 ± 11.43	Ref	54.20 ± 7.79	Ref	49.79 ± 9.53	Ref	48.83 ± 8.73	Ref	42.36 ± 11.46	Ref
5th quintile (*n* = 57)	52.98 ± 10.04	1.99 (−1.48, 5.46)	55.26 ± 6.15	0.43 (−1.87, 2.74)	51.95 ± 8.40	1.86 (−0.95, 4.66)	51.58 ± 7.27	2.67 (−0.04, 5.38)	44.56 ± 10.10	2.00 (−1.53, 5.53)
**TPOAb**										
Negative (*n* = 205)	52.02 ± 10.48	Ref	54.29 ± 7.06	Ref	50.62 ± 8.83	Ref	50.17 ± 8.02	Ref	43.51 ± 11.01	Ref
Positive (*n* = 29)	52.59 ± 11.15	0.52 (−3.74, 4.78)	54.48 ± 10.03	0.98 (−1.90, 3.85)	50.69 ± 9.89	0.45 (−3.04, 3.93)	47.76 ± 10.99	−2.29 (−5.69, 1.10)	42.59 ± 12.79	−0.56 (−4.96, 3.84)

*FT4, free thyroxine; FT3, free triiodothyronine; TSH, thyroid-stimulating hormone; TPOAb, thyroid peroxidase antibody. TPOAb positive: ≥5.61 IU/ml, CI: confidence interval.*

*The regression coefficients were adjusted for maternal age (<30, 30–34, ≥35 years), education (high school or lower, college/university), birth weight for gestational age (SGA, AGA, LGA), infant feeding pattern during the first 6 months of life (exclusive breast-feeding, mixed feeding, formula feeding), infant passive smoking in the first 2 years (yes, no), and infant age at ASQ-3 assessment.*

^*^p < 0.05; ^**^p < 0.01.

For the girls, compared to those with cord serum FT3 in the middle 60%, both those with FT3 in the highest and lowest quintiles had higher scores in the communication domain, with 3.06 (0.24, 5.88) points and 3.26 (0.46, 6.06), respectively, increase in points (Table 4).

**Table 4:** Associations between cord blood thyroid hormones and ASQ-3 scores in girls at 24 months of age.

**Thyroid related hormones**	**Communication**		**Gross motor**		**Fine motor**		**Problem solving**		**Personal-social**	
	**Mean±SD**	**Adjusted**	**Mean±SD**	**Adjusted**	**Mean±SD**	**Adjusted**	**Mean±SD**	**Adjusted**	**Mean±SD**	**Adjusted**
		**β (95% CI)**		**β (95% CI)**		**β (95% CI)**		**β (95% CI)**		**β (95% CI)**
**FT4 (pmol/L)**										
1st quintile (*n* = 47)	55.21 ± 9.44	−0.31 (−3.08, 2.45)	53.30 ± 8.42	−2.20 (−5.01, 0.62)	51.81 ± 7.55	−0.26 (−3.03, 2.51)	51.49 ± 8.00	−0.05 (−2.92, 2.82)	46.28 ± 11.58	−1.99 (−5.49, 1.50)
Mid 3 quintiles (*n* = 123)	55.73 ± 7.75	Ref	55.56 ± 7.18	Ref	51.87 ± 8.08	Ref	51.59 ± 8.38	Ref	48.37 ± 9.46	Ref
5th quintile (*n* = 45)	58.00 ± 5.05	2.00 (−0.79, 4.80)	53.78 ± 9.12	−1.67 (−4.51, 1.18)	51.78 ± 7.40	−0.95 (−3.74, 1.85)	51.67 ± 8.73	−0.08 (−2.97, 2.82)	49.22 ± 9.04	−0.03 (−3.56, 3.50)
**FT3 (pmol/L)**										
1st quintile (*n* = 43)	58.49 ± 3.19	3.26 (0.46, 6.06)*	55.33 ± 9.20	0.86 (−2.05, 3.77)	53.37 ± 7.05	1.39 (−1.45, 4.23)	53.26 ± 6.89	1.22 (−1.73, 4.16)	50.33 ± 8.34	3.03 (−0.54, 6.59)
Mid 3 quintiles (*n* = 133)	54.85 ± 9.02	Ref	54.36 ± 7.70	Ref	51.62 ± 7.75	Ref	51.32 ± 8.59	Ref	47.07 ± 10.32	Ref
5th quintile (*n* = 39)	57.69 ± 5.24	3.06 (0.24, 5.88)*	55.13 ± 7.30	0.78 (−2.14, 3.71)	50.90 ± 8.65	−0.68 (−3.54, 2.17)	50.64 ± 8.82	−0.18 (−3.14, 2.78)	49.10 ± 9.66	2.37 (−1.22, 5.96)
**TSH (mIU/L)**										
1st quintile (*n* = 43)	55.93 ± 8.54	−0.27 (−3.04, 2.50)	53.95 ± 7.91	−0.93 (−3.76, 1.90)	52.44 ± 7.67	0.10 (−2.66, 2.85)	52.79 ± 7.81	1.05 (−1.81, 3.91)	50.00 ± 10.06	1.18 (−2.30, 4.65)
Mid 3 quintiles (*n* = 140)	55.82 ± 7.99	Ref	54.96 ± 7.79	Ref	51.89 ± 7.71	Ref	51.32 ± 8.32	Ref	48.01 ± 9.88	Ref
5th quintile (*n* = 32)	57.50 ± 4.92	1.72 (−1.39, 4.84)	54.50 ± 8.71	−0.35 (−3.53, 2.84)	50.78 ± 8.43	−1.87 (−4.97, 1.23)	51.09 ± 9.13	−0.40 (−3.61, 2.82)	45.84 ± 9.48	−2.71 (−6.62, 1.20)
**TPOAb**										
Negative (*n* = 196)	55.97 ± 7.95	Ref	54.69 ± 8.04	Ref	52.07 ± 7.82	Ref	51.40 ± 8.61	Ref	47.95 ± 10.07	Ref
Positive (*n* = 19)	57.37 ± 4.82	0.93 (−3.00, 4.85)	54.74 ± 6.76	−1.04 (−5.05, 2.96)	49.47 ± 7.24	−2.18 (−6.08, 1.72)	53.42 ± 4.43	1.68 (−2.37, 5.72)	49.47 ± 7.80	1.60 (−3.34, 6.54)

*FT4, free thyroxine; FT3, free triiodothyronine; TSH, thyroid-stimulating hormone; TPOAb, thyroid peroxidase antibody. TPOAb positive: ≥5.61 IU/ml, CI, confidence interval.*

*The regression coefficients were adjusted with maternal age (<30, 30–34, ≥35 years), education (high school or lower, college/university), birth weight for gestational age (SGA, AGA, LGA), infant feeding pattern during the first 6 months of life (exclusive breast-feeding, mixed feeding, formula feeding), infant passive smoking in the first 2 years (yes, no), and infant age at ASQ-3 assessment.*

^*^p < 0.05.

There was no significant association between cord blood TSH and TPOAb positivity with ASQ-3 scores at 24 months of age in both boys and girls (Tables 3, 4).

## Discussion

In this birth cohort conducted in term-born infants of euthyroid mother, for boys, lower levels of cord blood FT4 was associated with lower ASQ-3 scores in the communication, fine motor, and personal-social domains, and higher FT3 levels was associated with higher scores in the personal-social domain in boys at age 24 months. These associations were not observed in the girls. Cord blood TSH and TPOAb positivity were not associated with ASQ-3 scores during infancy.

Our study showed that mild alterations in cord blood FT4 were associated with ASQ-3 scores in the boys but not in the girls. Few studies had explored sex dimorphisms in these associations in human population. Animal studies reported that in fetal brains of guinea pig, the mRNA expression of thyroid hormone receptors (TRα1 and β1) increased in male but decreased in female with hypothyroxinaemia (35). A sex-specific association was found between maternal thyroid dysfunction in early pregnancy and school performance in adolescent girls and boys (36). Maternal higher iodine intake during pregnancy was associated with higher risk of poorer psychomotor achievements in girls, but the risk was lower in boys (37).

We observed that low cord blood FT4 levels in boys of euthyroid mother were associated with suboptimal neurodevelopment in the communication, fine motor, and personal-social domains. There have been inconsistent findings on FT4 or T4 and neurodevelopment (6, 16–19). Some studies showed that lower FT4 or T4 levels were associated with poorer cognitive, verbal, and psychomotor development (6, 16, 17). In contrast, negative or no association between neonatal T4 and cognitive outcomes were also reported (18, 19). Before mid-gestation, maternal supply is the only source of fetal FT4, and there is lack of a compensatory regulation of cerebral enzyme type 2 deiodinase (D2) bioactivity (38) involved in the local conversion of T4 to active T3 in the brain (38). Therefore, lower maternal T4, even within the normal range, could lead to lower FT4 supply to fetus; thus, lower T3 is available for brain development (39). Neonatal T4 and T3 levels may partly reflect maternal T4 levels during gestation (40). The brain regions primarily affected by FT4 are the hippocampus, cortex, and cerebellum, involved in memory, learning, cognition, and motor abilities (41). Our findings suggest that even mild alternations in fetal thyroid hormone levels of euthyroid mother may affect neurodevelopment during infancy.

In this study, no associations were observed between cord blood serum TSH and ASQ-assessed neurodevelopment in boys or girls. This finding was consistent with previous studies (42). In traditional views, TSH could reflect mild and subclinical variations of thyroid hormones. This study was limited to term-born infants of euthyroid mothers. T3 is the bioactive form of thyroxine and essential for fetal cerebral cortex development (39). A substantial part of intracellular T3 supply in the brain depends on circulating T4 transport and local conversion by the D2 enzyme (43). A cross-sectional study on 4-year-old children found no association between serum FT3 concentration and mental or motor development (11). Few studies had examined cord blood T3 and infant neurodevelopment. A previous study on preterm infants found that a decrease between cord blood T3 and infant serum T3 levels at the end of the first week of life was associated with an increased risk of disturbed mental development at 9 months but not at 24 months of age (44). In this study, a “U”-shaped relationship was found between cord blood FT3 levels and scores in the communication domain in girls. Future confirmation studies are needed.

In general, relatively lower neonatal FT4 levels are associated with suboptimal performance in the communication, fine motor, and personal-social domains in boys. Brain structure and function affected by thyroid hormones may not be global but involve subregions (41). During gestation and the first 2 years of postnatal life, thyroid hormone availability in the brain is under a complex temporal and spatial regulation, exquisitely tailored by the iodothyronine deiodinase system; D2 generates T3 from T4, and the enzyme type 3 deiodinase (D3) protects brain regions from excessive T3 until differentiation is required (38). Thyroid hormone T3 also affects neurodevelopment through activation of the phosphatidylinositol 3-kinaseprotein kinase AKT(PI3K/AKT) pathway by affecting nitrergic innervation function (45–47).

It is assumed that TPOAb/thyroglobulin (TgAb) in newborns is of maternal origin, but there is limited information on the association between cord TPOAb/TgAb and neurodevelopment (18). We observed that there was no association between cord blood TPOAb positivity and infant neurodevelopment. Williams et al. also reported no association between the McCarthy score and cord TPOAb, but the Perceptual Performance subscale score was significantly lower in infants with cord TgAb positivity (18). The association between maternal TPOAb positivity and child IQ may partially depend on iodine status (48). A previous study has shown that term-born infants with positive thyroid antibodies had normal thyroid function (18). More studies with larger cohorts are needed to clarify the association between cord TPOAb/TgAb and neurodevelopment in infants.

Our study had some limitations. We did not measure maternal urinary iodine status, but iodine is known to be adequate in this study population because of ubiquitous consumption of iodine salt (49). The study participants are all Chinese, and the findings may not be generalizable to other populations.

## Conclusion

Mild alterations in thyroid hormones of newborns with maternal euthyroid may affect neurodevelopment during infancy in boys. The results of this study suggest the importance of optimal thyroid hormones status for neurodevelopment in early life.

## Data Availability Statement

The datasets generated for this study are available on request to the corresponding author.

## Ethics Statement

The studies involving human participants were reviewed and approved by Medical Ethics Committee of Xinhua Hospital, Shanghai Jiao Tong University School of Medicine. Written informed consent to participate in this study was provided by the participants' legal guardian/next of kin.

## Author Contributions

FO conceived, designed, conducted the study, analyzed and interpreted the data, and drafted and revised the manuscript. PF analyzed and interpreted the data and drafted the manuscript. Z-CL and JZ contributed to data interpretation and intensively revised the manuscript. YC, LS, WW, and ZL contributed in study conduct and critically revised the manuscript for important intellectual content. All the authors have reviewed and approved the final version of the manuscript as submitted and agree to be accountable for all aspects of the study.

## Funding

This study was supported by grants from the National Key R&D Program of China (Grant No. 2017YFE0124700), National Natural Science Foundation of China (Grant No. 81961128023), Shanghai Municipal Education Commission—Gaofeng Clinical Medicine (Grant No. 20152518), and Shanghai Municipal Health Commission (2020CXJQ01, GWV-10.1-XK07).

## Conflict of Interest

The authors declare that the research was conducted in the absence of any commercial or financial relationships that could be construed as a potential conflict of interest.

## Publisher's Note

All claims expressed in this article are solely those of the authors and do not necessarily represent those of their affiliated organizations, or those of the publisher, the editors and the reviewers. Any product that may be evaluated in this article, or claim that may be made by its manufacturer, is not guaranteed or endorsed by the publisher.

## References

[1] Chapman A K Farmer ZJ Mastrandrea LD Matlock KA. Neonatal thyroid function and disorders. Clin Obstetr Gynecol. (2019) 62:373–87. 10.1097/GRF.0000000000000434 31026231

[2] Patel J Landers K Li H Mortimer RH Richard K. Thyroid hormones and fetal neurological development. J Endocrinol. (2011) 209:1–8. 10.1530/JOE-10-0444 21212091

[3] Forhead AJ Fowden AL. Thyroid hormones in fetal growth and prepartum maturation. J Endocrinol. (2014) 221:R87–103. 10.1530/JOE-14-0025 24648121

[4] Ghassabian A Henrichs J Tiemeier H. Impact of mild thyroid hormone deficiency in pregnancy on cognitive function in children: lessons from the generation R study. Best Pract Res Clin Endocrinol Metab. (2014) 28:221–32. 10.1016/j.beem.2013.04.008 24629863

[5] Williams GR. Neurodevelopmental and neurophysiological actions of thyroid hormone. J Neuroendocrinol. (2008) 20:784–94. 10.1111/j.1365-2826.2008.01733.x 18601701

[6] Delahunty C Falconer S Hume R Jackson L Midgley P Mirfield M. Levels of neonatal thyroid hormone in preterm infants and neurodevelopmental outcome at 5 1/2 years: millennium cohort study. J Clin Endocrinol Metab. (2010) 95:4898–908. 10.1210/jc.2010-0743 20719832

[7] Wassie M M Smithers L G Zhou S J. Association between newborn thyroid-stimulating-hormone concentration and neurodevelopment and growth: a systematic review. Biol Trace Element Res. (2021). 10.1007/s12011-021-02665-7. [Epub ahead of print]. 33686634

[8] Freire C Ramos R Amaya E Fernandez MF Santiago-Fernandez P Lopez-Espinosa MJ. Newborn TSH concentration and its association with cognitive development in healthy boys. Eur J Endocrinol. (2010) 163:901–9. 10.1530/EJE-10-0495 20829366

[9] Perez-Lobato R Ramos R Arrebola J P Calvente I Ocon-Hernandez O Davila-Arias C. Thyroid status and its association with cognitive functioning in healthy boys at 10 years of age. Eur J Endocrinol. (2015) 172:129–39. 10.1530/EJE-14-0093 25394567

[10] Belcari F Placidi G Guzzetta A Tonacchera M Ciampi M Bartoli A. Thyroid-stimulating hormone levels in the first days of life and perinatal factors associated with sub-optimal neuromotor outcome in pre-term infants. J Endocrinol Invest. (2011) 34:e308–13. 10.3275/7795 21659794

[11] Alvarez-Pedrerol M Ribas-Fitó N Torrent M Julvez J Ferrer C Sunyer J. TSH concentration within the normal range is associated with cognitive function and ADHD symptoms in healthy preschoolers. Clin Endocrinol. (2007) 66:890–8. 10.1111/j.1365-2265.2007.02871.x 17535399

[12] Trumpff C De Schepper J Vanderfaeillie J Vercruysse N Van Oyen H Moreno-Reyes R. Neonatal thyroid-stimulating hormone concentration and psychomotor development at preschool age. Arch Dis Child. (2016) 101:1100–06. 10.1136/archdischild-2015-310006 27402733 PMC5256416

[13] Trumpff C De Schepper J Vanderfaeillie J Vercruysse N Van Oyen H Moreno-Reyes R. Thyroid-Stimulating hormone (TSH) concentration at birth in belgian neonates and cognitive development at preschool age. Nutrients. (2015) 7:9018–32. 10.3390/nu7115450 26540070 PMC4663578

[14] Trumpff C De Schepper J Vanderfaeillie J Vercruysse N Tafforeau J Van Oyen H. No association between elevated thyroid-stimulating hormone at birth and parent-reported problem behavior at preschool age. Front Endocrinol. (2016) 7:161. 10.3389/fendo.2016.00161 28066326 PMC5165276

[15] Lain SJ Bentley JP Wiley V Roberts CL Jack M Wilcken B. Association between borderline neonatal thyroid-stimulating hormone concentrations and educational and developmental outcomes: a population-based record-linkage study. Lancet Diabetes Endocrinol. (2016) 4:756–65. 10.1016/S2213-8587(16)30122-X 27453174

[16] van Wassenaer AG Briët J M van Baar A Smit BJ Tamminga P de Vijlder JJ. Free thyroxine levels during the first weeks of life and neurodevelopmental outcome until the age of 5 years in very preterm infants. Pediatrics. (2002) 110:534–9. 10.1542/peds.110.3.534 12205256

[17] Simic N Asztalos E V Rovet J. Impact of neonatal thyroid hormone insufficiency and medical morbidity on infant neurodevelopment and attention following preterm birth. Thyroid. (2009) 19:395–401. 10.1089/thy.2008.0282 19355829

[18] Williams FL Watson J Ogston SA Visser T J Hume R Willatts P. Maternal and umbilical cord levels of T4, FT4, TSH, TPOAb, and TgAb in term infants and neurodevelopmental outcome at 5.5 years. J Clin Endocrinol Metab. (2013) 98:829–38. 10.1210/jc.2012-3572 23322817

[19] Lain SJ Rifas-Shiman SL Pearce EN Nassar N Oken E. Neonatal thyroxine, maternal thyroid function, and cognition in mid-childhood in a US cohort. Mater Child Health J. (2020) 24:503–13. 10.1007/s10995-019-02867-5 31897929 PMC7083173

[20] Hollanders JJ Israëls J van der Pal SM Verkerk PH Rotteveel J Finken MJ. No association between transient hypothyroxinemia of prematurity and neurodevelopmental outcome in young adulthood. J Clin Endocrinol Metab. (2015) 100:4648–53. 10.1210/jc.2015-3078 26480285

[21] Soldin OP Lai S Lamm SH Mosee S. Lack of a relation between human neonatal thyroxine and pediatric neurobehavioral disorders. Thyroid. (2003) 13:193–8. 10.1089/105072503321319503 12699594 PMC3641764

[22] Oken E Braverman LE Platek D Mitchell ML Lee SL Pearce EN. Neonatal thyroxine, maternal thyroid function, and child cognition. J Clin Endocrinol Metab. (2009) 94:497–503. 10.1210/jc.2008-0936 19033373 PMC2646520

[23] van Wassenaer AG Kok JH de Vijlder JJ Briët JM Smit BJ Tamminga P. Effects of thyroxine supplementation on neurologic development in infants born at less than 30 weeks' gestation. N Engl J Med. (1997) 336:21–6. 10.1056/NEJM199701023360104 8970936

[24] Marchal JP Maurice-Stam H Ikelaar NA Klouwer FC Verhorstert KW Witteveen ME. Effects of early thyroxine treatment on development and growth at age 10.7 years: follow-up of a randomized placebo-controlled trial in children with Down's syndrome. J Clin Endocrinol Metab. (2014) 99:E2722–9. 10.1210/jc.2014-2849 25243574

[25] Reynolds JE Grohs MN Dewey D Lebel C. Global and regional white matter development in early childhood. NeuroImage. (2019) 196:49–58. 10.1016/j.neuroimage.2019.04.004 30959194

[26] Williams FL Mires GJ Barnett C Ogston SA van Toor H Visser TJ. Transient hypothyroxinemia in preterm infants: the role of cord sera thyroid hormone levels adjusted for prenatal and intrapartum factors. J Clin Endocrinol Metab. (2005) 90:4599–606. 10.1210/jc.2005-0214 15886240

[27] Ouyang F Tang N Zhang HJ Wang X Zhao S Wang W. Maternal urinary triclosan level, gestational diabetes mellitus and birth weight in Chinese women. Sci Total Environ. (2018) 626:451–7. 10.1016/j.scitotenv.2018.01.102 29353787 PMC5849787

[28] Wang X Ouyang F Feng L Wang X Liu Z Zhang J. Maternal urinary triclosan concentration in relation to maternal and neonatal thyroid hormone levels: a prospective study. Environ Health Perspect. (2017) 125:067017. 10.1289/EHP500 28669941 PMC5743753

[29] Squires J Bricker D. Ages & Stages Questionnaires®, Third Edition (ASQ®-3): A Parent-Completed Child Monitoring System. Baltimore, MD: Brookes Publishing Co., Inc (2009). 10.1037/t11523-000

[30] Bian X Yao G Squires J Wei M Chen CI Fang B. Studies of the norm and psychometric properties of ages and stages questionnaires in Shanghai Children. Chin J Pediatr. (2010) 48:492–496. 10.3760/cma.j.issn.0578-1310.2010.07.003 21055084

[31] Wei M Bian X Squires J Yao G Wang X Xie H. Studies of the norm and psychometrical properties of the ages and stages questionnaires, third edition, with a Chinese national sample. Chin J Pediatr. (2015) 53:913–8. 10.3760/cma.j.issn.0578-1310.2015.12.009 26887546

[32] Sirkka O Hof MH Vrijkotte T Abrahamse-Berkeveld M Halberstadt J Seidell J C. Feeding patterns and BMI trajectories during infancy: a multi-ethnic, prospective birth cohort. BMC Pediatr. (2021) 21:34. 10.1186/s12887-020-02456-4 33441111 PMC7805191

[33] Li Z Rong Z Shulian Z Wenjing S Weili Y Xiaoli W. Chinese neonatal birth weight curve for different gestational age. Chin J Pediatr. (2015) 53:97–103. 10.3760/cma.j.issn.0578-1310.2015.02.007 25876683

[34] Fan P Luo ZC Tang N Wang W Liu Z Zhang J. Advanced maternal age, mode of delivery, and thyroid hormone levels in Chinese newborns. Front Endocrinol. (2019) 10:913. 10.3389/fendo.2019.00913 31998241 PMC6966407

[35] Chan SY Andrews MH Lingas R McCabe CJ Franklyn JA Kilby MD. Maternal nutrient deprivation induces sex-specific changes in thyroid hormone receptor and deiodinase expression in the fetal guinea pig brain. J Physiol. (2005) 566 (Pt. 2):467–80. 10.1113/jphysiol.2005.084673 PMC1464754 15878952

[36] Pakkila F Mannisto T Hartikainen AL Ruokonen A Surcel HM Bloigu A. Maternal and child's thyroid function and child's intellect and scholastic performance. Thyroid. (2015) 25:1363–74. 10.1089/thy.2015.0197 26438036 PMC4684651

[37] Murcia M Rebagliato M Iñiguez C Lopez-Espinosa M J Estarlich M Plaza B. Effect of iodine supplementation during pregnancy on infant neurodevelopment at 1 year of age. Am J Epidemiol. (2011) 173:804–12. 10.1093/aje/kwq424 21385833

[38] de Escobar GM Obregón MJ del Rey FE. Maternal thyroid hormones early in pregnancy and fetal brain development. Best Pract Res Clin Endocrinol Metab. (2004) 18:225–48. 10.1016/j.beem.2004.03.012 15157838

[39] Morreale de Escobar G Obregon MJ Escobar del Rey F. Role of thyroid hormone during early brain development. Eur J Endocrinol. (2004) 151 (Suppl. 3):U25–37. 10.1530/eje.0.151u025 15554884

[40] Medici M de Rijke YB Peeters RP Visser W de Muinck Keizer-Schrama SM Jaddoe VV. Maternal early pregnancy and newborn thyroid hormone parameters: the generation R study. J Clin Endocrinol Metab. (2012) 97:646–52. 10.1210/jc.2011-2398 22162477

[41] Min H Dong J Wang Y Wang Y Teng W Xi Q. Maternal hypothyroxinemia-induced neurodevelopmental impairments in the progeny. Mol Neurobiol. (2016) 53:1613–24. 10.1007/s12035-015-9101-x 25666160

[42] Azizi F Afkhami M Sarshar A Nafarabadi M. Effects of transient neonatal hyperthyrotropinemia on intellectual quotient and psychomotor performance. Int Vitam Nutr. (2001) 71:70–3. 10.1024/0300-9831.71.1.70 11276926

[43] Bernal J Guadaño-Ferraz A Morte B. Perspectives in the study of thyroid hormone action on brain development and function. Thyroid. (2003) 13:1005–12. 10.1089/105072503770867174 14651784

[44] Eerdekens A Naulaers G Ortibus E Verhaeghe J Langouche L Vanhole C. Evolution of circulating thyroid hormone levels in preterm infants during the first week of life: perinatal influences and impact on neurodevelopment. J Pediatr Endocrinol Metab. (2019) 32:597–606. 10.1515/jpem-2018-0537 31112508

[45] Hiroi Y Kim HH Ying H Furuya F Huang Z Simoncini T. Rapid nongenomic actions of thyroid hormone. Proc Natl Acad Sci USA. (2006) 103:14104–9. 10.1073/pnas.0601600103 16966610 PMC1599919

[46] Llevenes P Balfagon G Blanco-Rivero J. Thyroid hormones affect nitrergic innervation function in rat mesenteric artery: role of the PI3K/AKT pathway. Vascul Pharmacol. (2018) 108:36–45. 10.1016/j.vph.2018.05.001 29751093

[47] Calabrese V Copani A Testa D Ravagna A Spadaro F Tendi E. Nitric oxide synthase induction in astroglial cell cultures: effect on heat shock protein 70 synthesis and oxidant/antioxidant balance. J Neurosci Res. (2000) 60:613–22. 10.1002/(SICI)1097-4547(20000601)60:5<613::AID-JNR6>3.0.CO;2-8 10820432

[48] Derakhshan A Korevaar TIM Taylor PN Levie D Guxens M Jaddoe VWV. The association of maternal thyroid autoimmunity during pregnancy with child IQ. J Clin Endocrinol Metab. (2018) 103:3729–3736. 10.1210/jc.2018-00743 30020468

[49] Wang Z Liu P Su X Zou S Song J Liu S. A comparison of iodine status in children and pregnant women after a policy change in the iodized salt standard in Shanghai, China. Biol Trace Element Res. (2018) 185:275–81. 10.1007/s12011-018-1257-6 29541993

