# Supplementary material for: Cord Blood Thyroid Hormones and Neurodevelopment in 2-Year-Old Boys and Girls
Source: Front Nutr. 2021 Dec 20;8:773965. doi: 10.3389/fnut.2021.773965 (PMC8720755; doi:10.3389/fnut.2021.773965)
Supplement: Supplementary file 1 [file Table_1.docx]

**Supplementary table 1**. Cord blood FT4, FT3, TSH level by quintiles and positive TPOAb proportion in vaginal and cesarean section deliveries

| Thyroid related hormones | Vaginal | | |  | Cesarean section | | |
| --- | --- | --- | --- | --- | --- | --- | --- |
|  | N | Mean$\pm$SD | Range |  | N | Mean$\pm$SD | Range |
| FT4 (pmol/L) |  |  |  |  |  |  |  |
| 1^st^ Quintile | 22 | 11.56$\pm$0.94 | 7.97-12.18 |  | 69 | 11.35$\pm$1.72 | <LOD-12.39 |
| 2^nd^ Quintile | 22 | 12.45$\pm$0.15 | 12.19-12.65 |  | 71 | 12.75$\pm$0.20 | 12.40-13.03 |
| 3^rd^ Quintile | 21 | 12.92$\pm$0.17 | 12.66-13.16 |  | 67 | 13.36$\pm$0.19 | 13.04-13.65 |
| 4^th^ Quintile | 23 | 13.55$\pm$0.29 | 13.19-14.12 |  | 69 | 14.12$\pm$0.27 | 13.66-14.59 |
| 5^th^ Quintile | 20 | 14.87$\pm$0.71 | 14.14-16.75 |  | 68 | 15.52$\pm$0.79 | 14.61-17.43 |
| FT3 (pmol/L) |  |  |  |  |  |  |  |
| 1^st^ Quintile | 22 | -- | <LOD-1.54 |  | 70 | -- | <LOD-1.79 |
| 2^nd^ Quintile | 22 | 1.69$\pm$0.09 | 1.57-1.83 |  | 68 | 1.94$\pm$0.08 | 1.80-2.04 |
| 3^rd^ Quintile | 21 | 1.94$\pm$0.07 | 1.84-2.04 |  | 68 | 2.14$\pm$0.06 | 2.05-2.22 |
| 4^th^ Quintile | 22 | 2.24$\pm$0.09 | 2.08-2.38 |  | 69 | 2.35$\pm$0.07 | 2.23-2.45 |
| 5^th^ Quintile | 21 | 2.64$\pm$0.19 | 2.43-3.24 |  | 66 | 2.89$\pm$1.57 | 2.46-15.19 |
| TSH (mIU/L) |  |  |  |  |  |  |  |
| 1^st^ Quintile | 22 | 3.87$\pm$0.58 | 1.99-4.44 |  | 69 | 3.09$\pm$0.40 | 1.74-3.55 |
| 2^nd^ Quintile | 22 | 5.46$\pm$0.56 | 4.55-6.52 |  | 69 | 3.93$\pm$0.21 | 3.56-4.23 |
| 3^rd^ Quintile | 21 | 7.30$\pm$0.39 | 6.55-7.81 |  | 69 | 4.64$\pm$0.25 | 4.24-5.05 |
| 4^th^ Quintile | 22 | 9.33$\pm$1.09 | 7.95-11.84 |  | 69 | 5.61$\pm$0.39 | 5.05-6.52 |
| 5^th^ Quintile | 21 | 19.41$\pm$7.31 | 11.86-44.93 |  | 68 | 9.28$\pm$3.69 | 6.54-28.87 |
| TPOAb (positive) | 10 (9.3%) | -- | -- |  | 38 (11.1%) | -- | -- |

FT4, free thyroxine; FT3, free triiodothyronine; TSH, thyroid stimulating hormone; TPOAb, thyroid peroxidase antibody.

The limit of detection (LOD) of FT4 is 5.15 pmol/L; the LOD of FT3 is 1.54 pmol/L; TPOAb positive: ≥5.61 IU/mL.

Sample size varied from 449 to 452 due to missing values.
